# Cost-effectiveness of adding Sativex® spray to spasticity care in Belgium: using bootstrapping instead of Monte Carlo simulation for probabilistic sensitivity analyses

**DOI:** 10.1007/s10198-021-01285-1

**Published:** 2021-04-20

**Authors:** Mark Oppe, Daniela Ortín-Sulbarán, Carlos Vila Silván, Anabel Estévez-Carrillo, Juan M. Ramos-Goñi

**Affiliations:** 1 Axentiva Solutions, S.L., C/Muntaner, 200 4º 5ª, 08036 Barcelona, Spain; 2 Almirall, Barcelona, Spain

**Keywords:** Cost–utility analysis, Probabilistic sensitivity analysis, Bootstrapping, Multiple sclerosis, Cannabinoids, I18, I19

## Abstract

**Background:**

Uncertainty in model-based cost-utility analyses is commonly assessed in a probabilistic sensitivity analysis. Model parameters are implemented as distributions and values are sampled from these distributions in a Monte Carlo simulation. Bootstrapping is an alternative method that requires fewer assumptions and incorporates correlations between model parameters.

**Methods:**

A Markov model-based cost–utility analysis comparing oromucosal spray containing delta-9-tetrahidrocannabinol + cannabidiol (Sativex®, nabiximols) plus standard care versus standard spasticity care alone in the management of multiple sclerosis spasticity was performed over a 5-year time horizon from the Belgian healthcare payer perspective. The probabilistic sensitivity analysis was implemented using a bootstrap approach to ensure that the correlations present in the source clinical trial data were incorporated in the uncertainty estimates.

**Results:**

Adding Sativex® spray to standard care was found to dominate standard spasticity care alone, with cost savings of €6,068 and a quality-adjusted life year gain of 0.145 per patient over the 5-year analysis. The probability of dominance increased from 29% in the first year to 94% in the fifth year, with the probability of QALY gains in excess of 99% for all years considered.

**Conclusions:**

Adding Sativex® spray to spasticity care was found to dominate standard spasticity care alone in the Belgian healthcare setting. This study showed the use of bootstrapping techniques in a Markov model probabilistic sensitivity analysis instead of Monte Carlo simulations. Bootstrapping avoided the need to make distributional assumptions and allowed the incorporation of correlating structures present in the original clinical trial data in the uncertainty assessment.

**Supplementary Information:**

The online version contains supplementary material available at 10.1007/s10198-021-01285-1.

## Introduction

Cost-effectiveness analysis (CEA) of new and current health services and technologies has become key in decision-making and health policy [1, 2]. Cost–utility analysis (CUA), a form of CEA that uses Quality-Adjusted Life Years (QALYs) as a measure of effectiveness, takes into account the value that individuals assign to health states [3–6]. Such evaluations provide key information to guide resource allocation considering the social perspective [1, 4–6]. One of the most common types of models used for CEA/CUA are Markov models. In these models, uncertainty around the cost-effectiveness results is commonly assessed using probabilistic sensitivity analyses (PSA). For model-based CEA, the PSA is usually implemented using Monte Carlo simulation. This requires that the model parameters are not implemented as point estimates, but as parametric distributions from which values are then sampled in the Monte Carlo simulation. Correlations between the parameters of the model are typically not included in the PSA, which can result in imprecise estimates of the uncertainty around the cost-effectiveness results.

In many cases, data at the individual patient level, such as those from a clinical trial, are used to calculate the parameters of a Markov model. The correlating structures present in these data can be incorporated in the PSA using bootstrapping techniques. Rather than sampling values from the distribution of each model parameter separately (as is done in a Monte Carlo simulation), values of the model parameters for the PSA can be calculated jointly for each bootstrap of the clinical trial data on which they are based. This ensures that any correlating structures present in these data sources are preserved and reflected in the estimates of the uncertainty from the PSA. In addition, the use of bootstrapping instead of Monte Carlo simulations avoids the need to make parametric assumptions about the distributions of the data used to populate the model. Therefore, the resulting estimates of the uncertainty of the CEA from the PSA reflect more accurately the uncertainty present in the clinical trial data that are used to calculate the parameters of the Markov model. In this study, we will demonstrate this in a CUA for a new cannabinoid-based approved medicine for the management of resistant multiple sclerosis (MS) spasticity (muscle rigidity and spasms) from a Belgian healthcare payer perspective.

MS is an autoimmune disease affecting the central nervous system [7, 8]. It is considered a high prevalence condition in Europe with a rate up to 83/100,000 inhabitants [9, 10]. One common symptom in MS patients is spasticity, present in up to 80% of patients as disease evolves [11], which is perceived as involuntary permanent activation of the muscles, often associated with spasms, pain and worsening of sleep, bladder function, mobility, and other MS spasticity associated symptoms [12]. MS reduces greatly the quality of life (QoL) of patients and interferes with the ability to work [12–14]. Furthermore, QoL diminishes as spasticity severity increases [15–18] and is one of the leading causes of distress in MS patients and caregivers [13, 19, 20]. Therefore, MS places a notorious economic burden in patients, family members, and health care systems [13, 21].

Management of MS aims to control and prevent the frequency and severity of the disease relapses, slowing down its progression [7, 22] and to minimize symptoms, including spasticity [23]. MS Standard-of-Care (SoC) implies multidisciplinary health care at once [7] as MS patients often need a combination of pharmacological treatment and physical therapy [23]. Sativex® (US adopted name nabiximols), a standardized compound of delta-9-tetrahydrocannabinol (THC) and cannabidiol (CBD) approved by health authorities in more than 20 countries, is indicated when patients become resistant to primary and/or secondary line of MS spasticity treatment [17, 24, 25]. The efficacy of this medication at reducing the severity of MS-related spasticity has been demonstrated in several randomized-controlled trials and observational studies [17, 26–31]. In 2012, two studies were published which assessed the cost-effectiveness of Sativex® as add-on therapy in comparison with oral anti-spasticity medicines alone for MS spasticity symptoms. The first, a study by Lu et al., was conducted in the UK setting [32]. The second, a study by Slof et al., considered the German and Spanish settings [33].

The Lu et al. study used a three-state Markov model with 4-week cycles from the National Health Service (NHS) perspective with a 5-year time horizon. The model applied a mean daily dose of 8.3 sprays obtained from a 12-week-long randomized clinical trial following the titration period and used this throughout the time horizon of the model [32]. The model resulted in an incremental cost-effectiveness ratio of £49,300. However, more recent data from the Mobility ImproVEment (MOVE) 2 study and an MS registry study in the UK indicated that patients adjusted their mean daily intake to 6.7 sprays/day after 3 months and down to 6.2 sprays/day after a year of ongoing treatment [34, 35] and the Sativex® daily dose was 4.2 sprays after a mean of 1.2 years of treatment [33, 36]. Therefore, the ICER was likely overestimated.

The study by Slof et al. [33] used a seven-state Markov model. They used the same length cycles and time horizon as Lu et al., but used dose per day figures that were in line with long-term use data. Their findings resulted in Sativex® being the dominant option in Spain, and cost-effective in Germany, with an ICER of €11,214 / QALY gained [33].

In 2015, another CUA was published by Slof et al. [34] using an updated version of the 2012 model to assess the cost-effectiveness of Sativex® in an Italian setting. The updates included a probabilistic sensitivity analysis (PSA) using Monte Carlo simulation. Using a 5-year time horizon, the 2015 model resulted in Sativex® being cost-effective in Italy with an ICER of €4,968/QALY gained. However, the uncertainty surrounding transition probabilities and resource use—both key model parameters—were not considered in the PSA. In addition, correlations between the model parameters were not considered in the PSA.

In the present study, the use of bootstrapping methodology instead of Monte Carlo simulation as a means of performing a PSA is presented. Moreover, the model uses the results from a Sativex® clinical trial that recently became available, the SAVANT trial [24]. In this trial, the efficacy of the medication as add-on treatment to SoC was assessed using a wash-out period to minimize carry-over effects, a method not applied in the previous studies. The uncertainty around the transition probabilities, the utilities, and the resource use were included in the bootstrap-based PSA. This allowed the assessment of the cost-effectiveness of Sativex® as add-on therapy for the treatment of MS spasticity in the Belgian setting, according to insights provided by the most recently available data and with more accurate assessment of the uncertainty than before.

## Methods

**Fig. 1:**
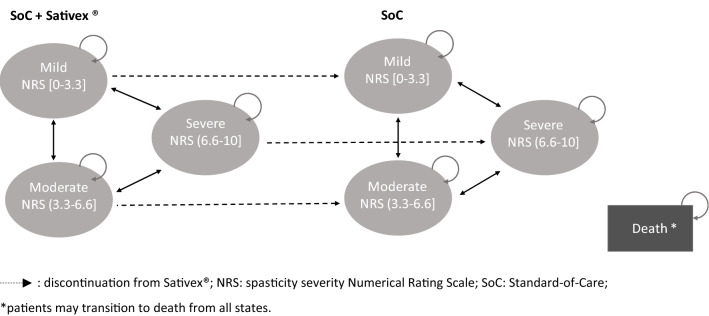
Schematic representation of the Markov model

A Markov model was used to evaluate the cost-effectiveness of standard-of-care with Sativex®(nabiximols) as adjuvant therapy compared to SoC alone. The model structure was based on the model published by Slof and colleagues in 2015 [34] and used the same 5-year time horizon (65 cycles). The model includes seven Markov states: three states for each arm related to severity of spasticity, and one state for death. The classification of severity of spasticity was based on MS spasticity Numerical Rating Scale (NRS) scores. NRS is a 10-point rating scale used to assess spasticity, ranging from 0 (no spasticity) to 10 (worst possible spasticity). Following past uses of the model, mild spasticity was defined as NRS score below 3.3, moderate spasticity as NRS score between 3.3 and 6.6, and severe spasticity as NRS score above 6.6 (see Fig. 1).

**Fig. 2:**
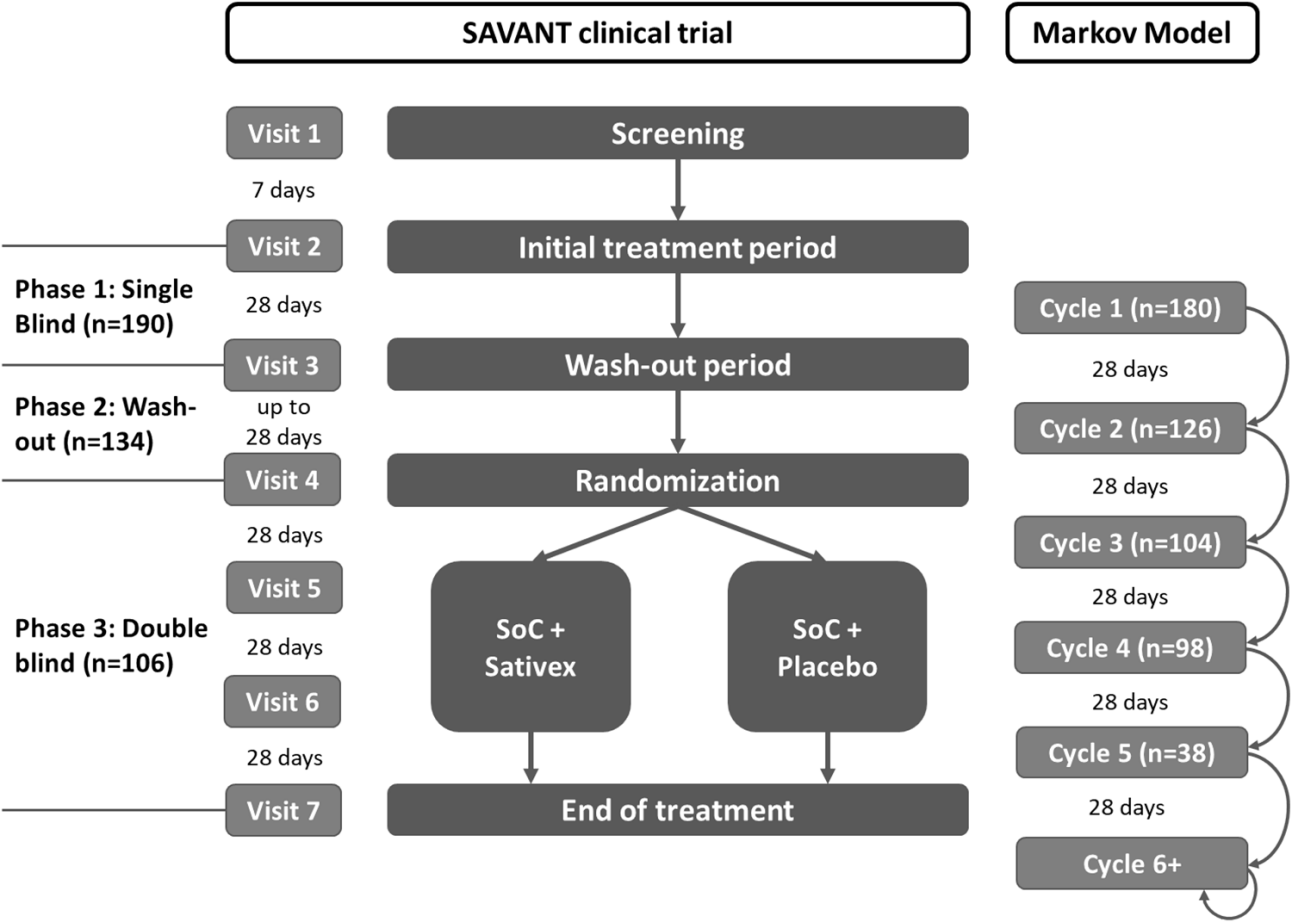
Overview of the design of the SAVANT trial and the cycles of the Markov model.* SoC* standard-of-care. Numbers between brackets indicate the total number of patients included in each phase of the trial, and available number of patients for calculating the transition probabilities for each cycle in the Markov model SoC standard-of-care

The main source of effectiveness data inputs in the model was the recent Sativex® SAVANT clinical trial (EudraCT Number: 2015-004451-40) [24]. The length of the cycles of the Markov model was 28 days to closely match the design of the clinical trial, facilitating the use of the bootstrap technique on the patient-level data in the PSA. The primary objective of the SAVANT trial was to evaluate the efficacy of Sativex® as add-on therapy compared to further optimized standard antispastic therapy in subjects with moderate-to-severe spasticity due to MS who had not gained adequate relief through two optimized standard antispastic drugs. The trial consisted of three phases after the initial screening period: a single-blinded trial period phase, a wash-out phase, and a double-blinded phase (see Fig. 2).

After initial screening (visit 1), 190 eligible patients entered the 4-week single-blinded trial period phase (visit 2) where every patient received Sativex® treatment in addition to SoC. Patients uptitrated the medication to their optimal efficacy dose (max. dose allowed 12 sprays per day) as an add-on to their standard antispastic medication, until they achieved symptom relief. During the single-blinded phase, 134 patients were identified as early responders (improvement of spasticity NRS score ≥ 20% versus baseline) and subsequently entered the wash-out phase. In the wash-out phase (visit 3), all initial responders continued to receive their underlying standard anti-spasticity medication but not Sativex®. The wash-out phase lasted until the MS spasticity NRS score was reduced by at least 80%. Patients who did not achieve this reduction in 4 weeks were withdrawn from the study. 106 patients fulfilled these requirements and entered the subsequent double-blinded phase (visits 4–7). Patients were randomly assigned to a 12-week treatment period with either Sativex® as add-on to the standard underlying antispastic medications (*n* = 53), or placebo as add-on (*n* = 53). All patients were asked to keep daily diaries between visits, which included self assessments of their spasticity using the NRS. Quality of life of patients was assessed using the SF-36 at each visit. Further details of the clinical trial can be found elsewhere [24].

### Transition probabilities

The transition probabilities between the mild, moderate, and severe states for the SoC + Sativex® arm for cycles 1–5 were calculated using the data from the SAVANT trial. In our base case scenario, transition probabilities for cycle 6 and subsequent cycles were assumed to be the same as those for cycle 5. Therefore, it is assumed that patients in SoC + Sativex® arm maintained the active treatment effects after the end of the trial period. Indeed, recent published literature has shown a treatment effect for a year or even longer [29, 37].

Available number of patients for calculating the transition probabilities ranged from *n* = 180 in cycle 1 to *n* = 38 for cycle 5 (end of randomized period, see Fig. 2). Probabilities for discontinuation with Sativex® for cycles 2, 3, and 4 were based on the SAVANT trial at 3.8% for mild, moderate, and severe. The discontinuation probability for cycle 5 and subsequent cycles was 3.5% based on a relevant long-term Sativex® study [38].

Transition probabilities between the mild, moderate, and severe spasticity state for the SoC arm were based on the retrospective observational study by Arroyo on MS spasticity patients previous to Sativex® introduction [39], rather than on the SAVANT trial because of its much longer time span (> 2 years). We included a scenario where the transition probabilities for the SoC arm were based on the SAVANT trial in the sensitivity analyses (see below).

General population life tables were obtained from the Belgium statistical office (2017) and used to determine the transition probabilities to death. These increased with the age of the cohort as it progressed through the model, ranging from 0.020% for the first cycle (i.e., age of 51) to 0.035% for the last cycles, corresponding to the age of 56. The same probabilities were used for both arms of the model. The transition probabilities between the mild, moderate, and severe states for the SoC + Sativex® arm can be found in Table 1. An overview of all transition probabilities used, including treatment discontinuation and death, can be found in the online appendix.

**Table 1:** Transition probabilities for the SoC + Sativex® arm of the model, based on the SAVANT trial in the base case scenario (transition probabilities for treatment discontinuation and death not shown)

**Cycle 1**	Mild	Moderate	Severe	**Cycle 4**	Mild	Moderate	Severe
Mild	0.219	0.719	0.062	Mild	0.962	0	0
Moderate	0.094	0.734	0.172	Moderate	0.111	0.851	0
Severe	0.100	0.100	0.800	Severe	0	0	0.962
**Cycle 2**	Mild	Moderate	Severe	**Cycle 5**	Mild	Moderate	Severe
Mild	0.769	0.096	0.096	Mild	0.844	0.121	0
Moderate	0.100	0.731	0.130	Moderate	0.241	0.724	0
Severe	0.048	0.433	0.481	Severe	0	0	0.965
**Cycle 3**	Mild	Moderate	Severe	**Cycle 6+**	Mild	Moderate	Severe
Mild	0.962	0	0	Mild	0.844	0.121	0
Moderate	0.329	0.633	0	Moderate	0.241	0.724	0
Severe	0	0.240	0.721	Severe	0	0	0.965

### Costs

#### Sativex® costs

Gradual initial dosing of Sativex® (nabiximols), as requested in the up-titration present in the approved label, was assumed in the model. The mean observed dose during the single-blind phase of the SAVANT trial was not reported in the publication by Markovà et al. [24]. Therefore, for the first cycle (first month trial period), a mean of 6.9 sprays/day was used, obtained from Novotna et al. (2011) [17]. For cycles 2–5, the mean observed dose at the end of the single-blind phase of the SAVANT trial was used (7.7 sprays/day). The lower mean observed dosage at the end of the double-blind phase from the SAVANT trial was used for cycle 6 (7.3 sprays/day). This was then linearly decreased for each cycle to a dose of 6.2 sprays/day at cycle 14 (i.e., at the 1 year time horizon), based on long-term dosage found in the long-term MOVE 2 observational study [35]. The dosage for cycle 14 was also used for all subsequent cycles (i.e., cycles 15–65 corresponding to a time horizon of up to 5 years). The Sativex® price used in the model was the list price of €466.40 for a 3 × 10 ml vial pack, where each 10 ml vial contains 90 pulverizations.

#### Other costs

In addition to costs related to the use of Sativex®, there are other costs associated with the spasticity standard-of-care. It was assumed that there were no differences in resource use related to SoC between the two arms of the model. Resource use estimates related to SoC were obtained from a Delphi panel consisting of eight clinical experts in the field of MS spasticity from Belgium. Experts individually provided their estimates on the treatment regimens used for patients with mild, moderate, and severe spasticity. Included were drug therapy; surgical procedures; healthcare system visits (e.g., primary care/GP, emergency room); and tests and monitoring (e.g., MRI, hematology). Unit costs for 2019 were obtained from the Belgian National Institute for Health and Disability Insurance (RIZIV) and the Belgian Centre for Pharmacotherapeutic Information (BCFI). Where up-to-date information on costs could not be found, costs were updated to 2019 values using published Belgian inflation rates. Details of all items included in the utilization of resources, together with the unit costs, are summarized in the online appendix. Costs were discounted using a discount rate of 3.0%, in line with Belgian guidelines [40].

### Quality of life and utilities

QoL data in the SAVANT trial were collected using the SF-36 QoL questionnaire [24]. Utilities for mild, moderate, and severe MS spasticity used in the model could not be based directly on EQ-5D-3L observations and were therefore derived via mapping. First, the 12 SF-36 responses that form the SF-12 were selected, thereby changing SF-36 profiles into SF-12 profiles. Next, the response mapping algorithm developed by Gray et al. [41] was used to convert reported individual patient SF-12 profiles into EQ-5D-3L utilities. The EQ-5D-3L utilities were calculated using the European VAS value set [42]. Following this procedure, EQ-5D utilities for each of the reported SF-12 profiles were created. This resulted in mean utilities for mild spasticity equal to 0.594, for moderate spasticity equal to 0.509, and for severe spasticity equal to 0.499. QALYs were discounted using a discount rate of 1.5% per year, in line with Belgian guidelines.

### Sensitivity analyses

The probabilistic sensitivity analysis (PSA) of the Markov model was performed using 1,000 bootstraps. In a bootstrap procedure multiple “bootstrapped” data sets are generated based on an “original” data set, by resampling with replacement from the original data (with the same size as the original sample). Each of these bootstrapped samples resembles a repetition of the original study. The bootstrapped samples differ from one another because of the sampling with replacement. The clinical and QoL data from the SAVANT trial were included in the bootstrap procedure as well as the resource use data from the Delphi panel.

For the clinical trial data, the bootstrap was stratified to retain the number of patients in each treatment arm at each phase of the clinical trial. The data from the 190 patients at the start of the single-blind phase, the 53 patients in the SoC + active treatment arm of the double-blind phase, and the 53 patients in the SoC arm of the double-blind phase were bootstrapped separately. This stratification was done to maintain the structure of the trial in the bootstraps. Therefore, the correlations between transitions probabilities between the three health states (mild, moderate, and severe) and the utilities of these health states that were stated within clinical trial data were all included in our PSA.

The bootstrap procedure incorporated the variability between SF-12 states reported by patients in the same Markov state. The mapped utilities for each of the SF-12 health states were included in our data as point estimates, since the uncertainty due to applying the mapping algorithm is minimal compared to the variability between SF-12 states reported by patients in the same Markov state [43].

The Delphi panel data on use of resources were included in the bootstrap procedure. This allowed potential differences in expert response characteristics and group bias to be reflected in the uncertainty estimates of the PSA. Following the same line of reasoning as for the utilities, the uncertainty surrounding the unit costs were not included in the PSA as they can be expected to be minimal compared to the uncertainty due to differences in resource use as provided by the Delphi panel.

The patient-level data from the SAVANT trial were used as is. Missing data were not imputed, because this would impact the estimates of the uncertainty of the data. Given the adherence to the trial protocol, a bias due to missing values was not expected . The data obtained from the Delphi panel did not contain any missing values. The algorithm that was followed for the bootstrapping procedure is specified in Box 1.

**Box 1:** Algorithm used for bootstrapping

Step 1:	Draw a random sample (with replacement) of the 53 patients from the SAVANT data for the SoC + Sativex arm of the randomisation period (phase 3 of the trial)
	Draw a random sample (with replacement) of the 53 patients from the SAVANT data for the for the SoC + Placebo arm of the randomisation period (phase 3 of the trial)
	Draw a random sample (with replacement) of the remaining patients from the SAVANT data that only completed phase 1 and 2 of the trial
Step 2:	Combine the 3 bootstrapped samples from step 1 and calculate the transition probabilities and utilities
Step 3:	Draw a random sample (with replacement) of the 8 Delphi panel members for resource use estimates
Step 4:	Calculate costs based on the resource use estimates obtained from step 3
Step 5:	Calculate and record the total costs and QALYs for both arms for the time horizon of the model
Step 6:	Repeat 1,000 times to obtain the probabilistic results

*SoC* standard of care; *SAVANT* Sativex as Add-on therapy vs further optimized first-line antispastics clinical trial; *QALYs* quality adjusted life years

**Fig. 3:**
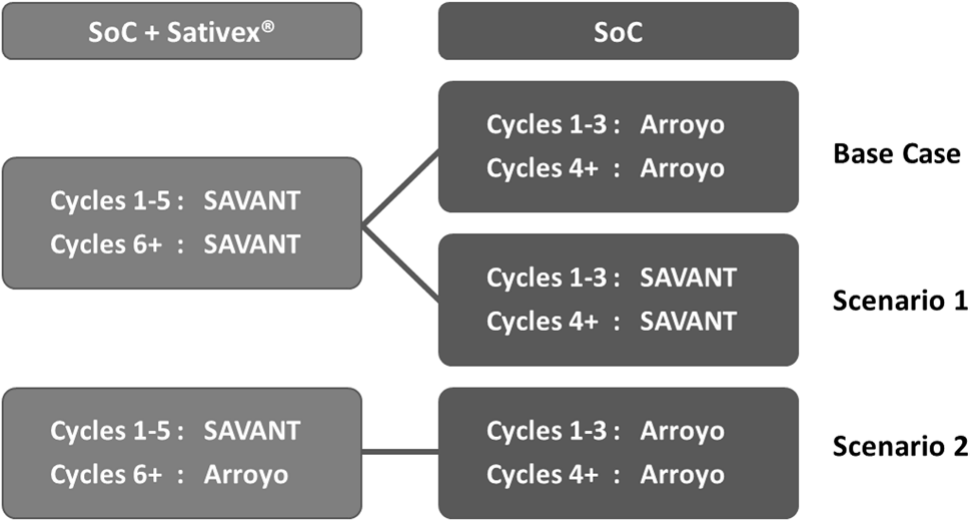
Source of the data used to determine the transition probabilities for the three scenarios. *SoC* standard of care; *SAVANT* Sativex as Add-on therapy Vs. further optimized first-line ANTispastics clinical trial; *Arroyo* retrospective observational study by Arroyo et al.

Since different sources of efficacy evidence were available, two scenarios were explored in addition to the base case analysis (see Fig. 3). In the base case scenario, transition probabilities between the mild, moderate, and severe state for the SoC arm were based on the long-term retrospective observational study by Arroyo et al. [39]. Scenario 1 explored instead the impact of calculating the SoC arm transition probabilities from the SAVANT trial data, meaning that in scenario 1, all efficacy data are based on the SAVANT trial. As the trial data were used, the bootstrapping procedure used for the PSA in scenario 1 also included the calculations for the transition probabilities of the SoC arm.

For scenario 2, patients in the SoC + Sativex® arm were assumed to have no further beneficial effect of the active medication following the clinical trial (i.e., after the 5th cycle), even though they still receive Sativex® treatment. That is, transition probabilities for cycle 6 and subsequent cycles were based on the long-term retrospective observational study by Arroyo [39] and, therefore, the same as in the SoC arm. This is a conservative scenario, since patients in the SoC + Sativex® arm after the end of the trial (from cycle 6 +) still incur the costs related to Sativex®, but no longer have any treatment benefits related to Sativex®. This scenario uses the least amount of data from the SAVANT trial.

The model implementation in MS Excel was evaluated using a thorough QA/QC evaluation of the existing model, including the model structure, the assumptions, calculations, and the programming.

## Results

**Fig. 4:**
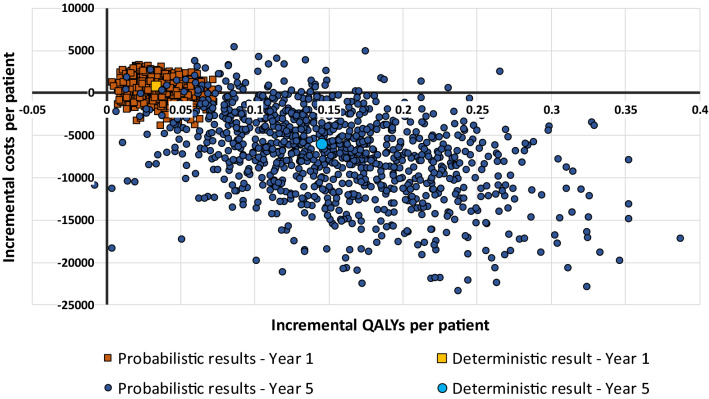
Cost-effectiveness plane for the base case model using time horizons of 1 and 5 years

Our base case resulted in SoC + Sativex® (nabiximols) being the dominant strategy (i.e., cost-saving while QALY gaining) for time horizons of 2 or more years, with ICERs between €22,187 for a 1-year time horizon decreasing to negative €41,942 for a 5-year time horizon (Table 2). The results from the PSA showed that the probability that SoC + Sativex® is dominant compared to SoC alone varied from 28.9% for a time horizon of 1 year to 94.1% for a time horizon of 5 years. The probability of QALY gains for patients was over 99% for all 5 time horizons. The cost-effectiveness plane showing the uncertainty surrounding the incremental costs and QALYs for the 1-year time horizon and the 5-year time horizon is shown in Fig. 4. Both additional conservative control scenarios (one using SAVANT trial data for the SoC arm transition probabilities and one assuming no further beneficial effect of the active medication after the 5th cycle for scenario 2) were no longer the dominant treatment option. However, both scenarios did result in ICERs below €25,000/QALY for time horizons of 2 years or longer (see Table 2).

**Table 2:** Incremental costs per patient, Incremental QALYs per patient, ICERs, and the probability that SoC + Sativex® is the dominant treatment option for the three scenarios

Time horizon	Incremental costs		Incremental QALYs		ICER		Dominance probability (%)
Base case							
1 year	€ 759	[- € 1,889, € 2,434]	0.034	[0.01, 0.06]	22,187	[-€63,201, €116,936]	29
2 years	- € 746	[- € 7,011, € 2,866]	0.072	[0.02, 0.14]	− 10,387	[-€106,280, € 75,901]	67
3 years	- € 2,610	[- € 11,304, € 2,512]	0.103	[0.03, 0.20]	− 25,417	[-€ 144,701, € 44,239]	82
4 years	- € 4,440	[- € 15,798, € 1,939]	0.127	[0.03, 0.24]	− 35,051	[-€ 145,330, € 24,614]	89
5 years	- € 6,068	[- € 18,833, € 1,442]	0.145	[0.04, 0.28]	− 41,942	[-€ 141,444, € 19,045]	94
Scenario 1							
1 year	€ 1,367	[- € 1,297, € 3,149]	0.018	[0.00, 0.04]	75,658	[-€ 514,022, € 874,758]	13
2 years	€ 940	[- € 4,628, € 4,494]	0.039	[- 0.01, 0.09]	24,300	[-€ 381,126, € 497,073]	34
3 years	€ 518	[- € 7,023, € 5,728]	0.052	[- 0.02, 0.13]	9,976	[-€ 581,595, € 637,440]	39
4 years	€ 402	[- € 10,046, € 6,992]	0.058	[- 0.04, 0.16]	6,979	[-€ 536,674, € 762,140]	46
5 years	€ 688	[- € 11,394, € 8,589]	0.057	[- 0.05, 0.17]	12,178	[-€ 704,778, €834,316]	43
Scenario 2							
1 year	€ 1,156	[- € 1,399, € 2,527]	0.029	[0.01, 0.05]	40,208	[-€ 45,976, € 138,371]	18
2 years	€ 1,001	[- € 3,520, € 3,618]	0.051	[0.02, 0.09]	19,697	[-€ 72,420, € 107,989]	33
3 years	€ 764	[- € 6,465, € 4,551]	0.055	[0.03, 0.11]	11,788	[-€ 80,727, € 93,624]	42
4 years	€ 510	[- € 5,375, € 5,293]	0.074	[0.03, 0.13]	6,921	[-€ 106,279, € 85,046]	46
5 years	€ 270	[- € 7,175, € 4,855]	0.079	[0.03, 0.14]	3,401	[-€ 102,361, €88,701]	49

Numbers between brackets indicate the 95% confidence intervals

## Discussion

In this study, we adapted an existing Markov model to assess cost-effectiveness of adding Sativex® spray to spasticity care to the Belgian setting. Markov models are commonly used for determining cost-effectiveness. Estimates of uncertainty around the cost-effectiveness figures are generally included in these models using probabilistic sensitivity analyses. In trial-based economic evaluations, bootstrapping is commonly used to obtain these estimates of uncertainty (see for example [44–46]); however, this is not the case when Markov models are used. For Markov models, in most cases, the PSA consists of a Monte Carlo simulation sampling values from model parameters implemented as different distributions. In this study, the uncertainty around the model parameters (and consequently around the obtained costs, QALYs, and ICERs) was estimated by means of bootstrapping techniques applied to the source data: the clinical trial data for the transition probabilities and utility estimates, and the resource use panel for the cost estimates. Note that the new sources of data (i.e., the SAVANT trial data and the Belgium specific model inputs) mostly impact the mean values of the model outcomes, whereas the bootstrapping procedure impacts the estimates of uncertainty. One advantage of bootstrapping compared to Monte Carlo simulation is that correlations (in our case between transition probabilities and utilities) are taken into account. Incorporating the correlation between model parameters can in principle also be done for Monte Carlo simulation using Cholesky Decomposition. However, that is only applicable to parameters with normal distributions. Given that in Markov models, the model parameters are typically not normal distributions, but, e.g., Dirichlet for probabilities, including the correlations properly is not feasible in most cases. This also highlights the second benefit of bootstrapping, namely that distributional assumptions can be avoided.

The transition probabilities and utilities used in the model were based on to the latest available sources of data for the active medication, using the recent SAVANT trial. Resource use estimates and unit costs from the Belgian healthcare public system perspective were used. Results showed that in the Belgian framework with a 5-year time horizon, adding Sativex® (nabiximols) to standard of care for the treatment of MS-related spasticity results in cost savings of €6,068 per patient with QALY gains of 0.145 per patient compared to standard of care treatment alone, being the dominant treatment strategy. The Bootstrapping PSA showed that the probability that Sativex® as add-on therapy to standard of care is the dominant strategy when using a 5-year time horizon at 94%. The analyses were carried out according to the Belgian HTA guidelines, which specify that no explicit discrete ICER threshold is used in Belgium [40].

Four previous cost-effectiveness analyses for Sativex® have been published. The study from the UK perspective from 2012 resulted in an ICER of £49,300 per QALY [32]. The study for Germany and Spain, also from 2012, resulted in an ICER of €11,214 per QALY for Germany, and in Sativex® being the dominant option in Spain [33]. The study for Italy from 2015 resulted in an ICER of €4,968 per QALY [34]. Our results, based on the Belgian setting and including the recent SAVANT trial as main source of our data, are similar to those found in Spain, and more favourable for Sativex®, when compared to the other three countries.

The Italian study included a PSA based on Monte Carlo simulations, but did not report the 95% confidence intervals surrounding the ICER [34]. Based on inspection of Fig. 3 of the paper (i.e., the cost-effectiveness acceptability curve), the 95% confidence interval surrounding the ICER can be estimated to be between €2,500 and €7,500 or between -50% and + 50% of the value of the ICER. Comparing those values with the results from this model showed that including uncertainty of the transition probabilities and resource use in the PSA leads to a much higher estimates of uncertainty surrounding the ICER. In addition, using bootstrapping in the PSA allowed for the incorporation of the correlation between the transition probabilities and utilities of the health states, which were based on the data from the same clinical trial. Additionally, there was no need to make any parametric assumptions with respect to the distributions for the transition probabilities, utilities, or resource use items. The latter were included in the PSA as a bootstrap over the Delphi panel.

The analyses of the two conservative scenarios demonstrated that the data source chosen for the transition probabilities impacted the results. However, in both cases, the ICER was below €25,000 from year 2 onwards. This implies that, although there is no dominance of Sativex® in these conservative scenarios, adding it as a treatment in MS patients is cost-effective.

A limitation of this study was that we did not include the uncertainty of all model parameters in the PSA. Uncertainty surrounding the transition probabilities based on the observational study by Arroyo et al. [39] (e.g., the SoC arm in the base case scenario) was obtained from the literature. Therefore, we could not include those in our bootstrap procedures for the PSA.

If the primary data are available, and this is the only source of data of that type, then bootstrapping should be preferred over Monte Carlo. If additional sources of data need to be included, they can be combined by incorporating them in the distributions used in Monte Carlo. Alternatively, if the additional sources of data allow this, the additional sources of data can be reverse engineered to match the format of the primary data (e.g., Kaplan–Meier curves can be reverse engineered into patient-level data). This would allow bootstrapping to be applied to the combined set. In cases where this is not possible (e.g., an NMA is required), the only option is to use Monte Carlo.

## Conclusion

The analyses showed that bootstrapping techniques can be used in the PSA of a Markov model, thereby properly incorporating correlations between model parameters and avoiding the need of making any parametric assumptions of the model inputs. In addition, the study showed that in a Belgium setting, under the modelled conditions, the use of Sativex® (nabiximols) as add-on therapy for resistant MS spasticity was found to be cost-saving, providing QALY gains compared to current standard-of-care treatment for resistant MS spasticity patients under safe conditions. Therefore, Sativex® should be considered as a valuable add-on treatment available for patients suffering from MS-related resistant spasticity.

## Supplementary Information

Below is the link to the electronic supplementary material.

Supplementary file1 (DOCX 50 KB)

## Data Availability

Not applicable.
