# Supplementary material for: Cost-effectiveness of adding Sativex® spray to spasticity care in Belgium: using bootstrapping instead of Monte Carlo simulation for probabilistic sensitivity analyses
Source: Eur J Health Econ. 2021 Apr 20;22(5):711–21. doi: 10.1007/s10198-021-01285-1 (PMC8214588; doi:10.1007/s10198-021-01285-1)
Supplement: Supplementary file 1 — Supplementary file1 (DOCX 50 KB) [file 10198_2021_1285_MOESM1_ESM.docx]

**Appendix A: Transition probabilities for the 3 scenarios used in the model**

**Table A1:** Transition probabilities for the Standard-of-Care + Sativex® arm of the model, based on the SAVANT trial used in the base case scenario. DC = discontinuation with Sativex

| **Cycle 1** | Mild | Moderate | Severe | DC Mild | DC Moderate | DC Severe | Death |
| --- | --- | --- | --- | --- | --- | --- | --- |
| Mild | 0.2187 | 0.7186 | 0.0625 | 0 | 0 | 0 | 0.0002 |
| Moderate | 0.0937 | 0.7342 | 0.1718 | 0 | 0 | 0 | 0.0002 |
| Severe | 0.1000 | 0.1000 | 0.7998 | 0 | 0 | 0 | 0.0002 |
| DC Mild | 0 | 0 | 0 | 0.9998 | 0 | 0 | 0.0002 |
| DC Moderate | 0 | 0 | 0 | 0 | 0.9998 | 0 | 0.0002 |
| DC Severe | 0 | 0 | 0 | 0 | 0 | 0.9998 | 0.0002 |
| Death | 0 | 0 | 0 | 0 | 0 | 0 | 1 |
|  |  |  |  |  |  |  |  |
| **Cycle 2** | Mild | Moderate | Severe | DC Mild | DC Moderate | DC Severe | Death |
| Mild | 0.7694 | 0.0962 | 0.0962 | 0.0380 | 0 | 0 | 0.0002 |
| Moderate | 0.1002 | 0.7314 | 0.1302 | 0 | 0.0380 | 0 | 0.0002 |
| Severe | 0.0481 | 0.4328 | 0.4809 | 0 | 0 | 0.0380 | 0.0002 |
| DC Mild | 0 | 0 | 0 | 0.9644 | 0.0204 | 0.0150 | 0.0002 |
| DC Moderate | 0 | 0 | 0 | 0.0000 | 0.9865 | 0.0133 | 0.0002 |
| DC Severe | 0 | 0 | 0 | 0 | 0.0057 | 0.9941 | 0.0002 |
| Death | 0 | 0 | 0 | 0 | 0 | 0 | 1 |
|  |  |  |  |  |  |  |  |
| **Cycle 3** | Mild | Moderate | Severe | DC Mild | DC Moderate | DC Severe | Death |
| Mild | 0.9618 | 0 | 0 | 0.0380 | 0 | 0 | 0.0002 |
| Moderate | 0.3290 | 0.6328 | 0 | 0 | 0.0380 | 0 | 0.0002 |
| Severe | 0 | 0.2404 | 0.7213 | 0 | 0 | 0.0380 | 0.0002 |
| DC Mild | 0 | 0 | 0 | 0.9644 | 0.0204 | 0.0150 | 0.0002 |
| DC Moderate | 0 | 0 | 0 | 0 | 0.9865 | 0.0133 | 0.0002 |
| DC Severe | 0 | 0 | 0 | 0 | 0.0057 | 0.9941 | 0.0002 |
| Death | 0 | 0 | 0 | 0 | 0 | 0 | 1 |
|  |  |  |  |  |  |  |  |
| **Cycle 4** | Mild | Moderate | Severe | DC Mild | DC Moderate | DC Severe | Death |
| Mild | 0.9618 | 0 | 0 | 0.0380 | 0 | 0 | 0.0002 |
| Moderate | 0.1110 | 0.8508 | 0 | 0 | 0.0380 | 0 | 0.0002 |
| Severe | 0 | 0 | 0.9618 | 0 | 0 | 0.0380 | 0.0002 |
| DC Mild | 0 | 0 | 0 | 0.9644 | 0.0204 | 0.0150 | 0.0002 |
| DC Moderate | 0 | 0 | 0 | 0 | 0.9865 | 0.0133 | 0.0002 |
| DC Severe | 0 | 0 | 0 | 0 | 0.0057 | 0.9941 | 0.0002 |
| Death | 0 | 0 | 0 | 0 | 0 | 0 | 1 |
|  |  |  |  |  |  |  |  |
| **Cycle 5** | Mild | Moderate | Severe | DC Mild | DC Moderate | DC Severe | Death |
| Mild | 0.8444 | 0.1206 | 0 | 0.0348 | 0 | 0 | 0.0002 |
| Moderate | 0.2412 | 0.7237 | 0 | 0 | 0.0348 | 0 | 0.0002 |
| Severe | 0 | 0 | 0.9650 | 0 | 0 | 0.0348 | 0.0002 |
| DC Mild | 0 | 0 | 0 | 0.9644 | 0.0204 | 0.0150 | 0.0002 |
| DC Moderate | 0 | 0 | 0 | 0 | 0.9865 | 0.0133 | 0.0002 |
| DC Severe | 0 | 0 | 0 | 0 | 0.0057 | 0.9941 | 0.0002 |
| Death | 0 | 0 | 0 | 0 | 0 | 0 | 1 |
|  |  |  |  |  |  |  |  |
| **Cycle 6** | Mild | Moderate | Severe | DC Mild | DC Moderate | DC Severe | Death |
| Mild | 0.8444 | 0.1206 | 0 | 0.0348 | 0 | 0 | 0.0002 |
| Moderate | 0.2412 | 0.7237 | 0 | 0 | 0.0348 | 0 | 0.0002 |
| Severe | 0 | 0 | 0.9650 | 0 | 0 | 0.0348 | 0.0002 |
| DC Mild | 0 | 0 | 0 | 0.9644 | 0.0204 | 0.0150 | 0.0002 |
| DC Moderate | 0 | 0 | 0 | 0 | 0.9865 | 0.0133 | 0.0002 |
| DC Severe | 0 | 0 | 0 | 0 | 0.0057 | 0.9941 | 0.0002 |
| Death | 0 | 0 | 0 | 0 | 0 | 0 | 1 |
|  |  |  |  |  |  |  |  |
| **Cycle 65** | Mild | Moderate | Severe | DC Mild | DC Moderate | DC Severe | Death |
| Mild | 0.8442 | 0.1206 | 0 | 0.0348 | 0 | 0 | 0.0003 |
| Moderate | 0.2412 | 0.7236 | 0 | 0 | 0.0348 | 0 | 0.0003 |
| Severe | 0 | 0 | 0.9649 | 0 | 0 | 0.0348 | 0.0003 |
| DC Mild | 0 | 0 | 0 | 0.9643 | 0.0204 | 0.0150 | 0.0003 |
| DC Moderate | 0 | 0 | 0 | 0 | 0.9863 | 0.0133 | 0.0003 |
| DC Severe | 0 | 0 | 0 | 0 | 0.0057 | 0.9939 | 0.0003 |
| Death | 0 | 0 | 0 | 0 | 0 | 0 | 1 |

**Table A2:** Transition probabilities for the Standard-of-Care arm of the model, based on the Arroyo et al. publication used in the base case scenario. DC = discontinuation with Sativex.

| **Cycle 1** | Mild | Moderate | Severe | DC Mild | DC Moderate | DC Severe | Death |
| --- | --- | --- | --- | --- | --- | --- | --- |
| Mild | 0.9644 | 0.0204 | 0.0150 | 0 | 0 | 0 | 0.0002 |
| Moderate | 0.0000 | 0.9865 | 0.0133 | 0 | 0 | 0 | 0.0002 |
| Severe | 0 | 0.0057 | 0.9941 | 0 | 0 | 0 | 0.0002 |
| DC Mild | 0 | 0 | 0 | 0.9998 | 0 | 0 | 0.0002 |
| DC Moderate | 0 | 0 | 0 | 0 | 0.9998 | 0 | 0.0002 |
| DC Severe | 0 | 0 | 0 | 0 | 0 | 0.9998 | 0.0002 |
| Death | 0 | 0 | 0 | 0 | 0 | 0 | 1 |
|  |  |  |  |  |  |  |  |
| **Cycle 2** | Mild | Moderate | Severe | DC Mild | DC Moderate | DC Severe | Death |
| Mild | 0.9644 | 0.0204 | 0.0150 | 0 | 0 | 0 | 0.0002 |
| Moderate | 0.0000 | 0.9865 | 0.0133 | 0 | 0 | 0 | 0.0002 |
| Severe | 0 | 0.0057 | 0.9941 | 0 | 0 | 0 | 0.0002 |
| DC Mild | 0 | 0 | 0 | 0.9998 | 0 | 0 | 0.0002 |
| DC Moderate | 0 | 0 | 0 | 0 | 0.9998 | 0 | 0.0002 |
| DC Severe | 0 | 0 | 0 | 0 | 0 | 0.9998 | 0.0002 |
| Death | 0 | 0 | 0 | 0 | 0 | 0 | 1 |
|  |  |  |  |  |  |  |  |
| **Cycle 3** | Mild | Moderate | Severe | DC Mild | DC Moderate | DC Severe | Death |
| Mild | 0.9644 | 0.0204 | 0.0150 | 0 | 0 | 0 | 0.0002 |
| Moderate | 0 | 0.9865 | 0.0133 | 0 | 0 | 0 | 0.0002 |
| Severe | 0 | 0.0057 | 0.9941 | 0 | 0 | 0 | 0.0002 |
| DC Mild | 0 | 0 | 0 | 0.9998 | 0 | 0 | 0.0002 |
| DC Moderate | 0 | 0 | 0 | 0 | 0.9998 | 0 | 0.0002 |
| DC Severe | 0 | 0 | 0 | 0 | 0 | 0.9998 | 0.0002 |
| Death | 0 | 0 | 0 | 0 | 0 | 0 | 1 |
|  |  |  |  |  |  |  |  |
| **Cycle 4** | Mild | Moderate | Severe | DC Mild | DC Moderate | DC Severe | Death |
| Mild | 0.9644 | 0.0204 | 0.0150 | 0 | 0 | 0 | 0.0002 |
| Moderate | 0 | 0.9865 | 0.0133 | 0 | 0 | 0 | 0.0002 |
| Severe | 0 | 0.0057 | 0.9941 | 0 | 0 | 0 | 0.0002 |
| DC Mild | 0 | 0 | 0 | 0.9998 | 0 | 0 | 0.0002 |
| DC Moderate | 0 | 0 | 0 | 0 | 0.9998 | 0 | 0.0002 |
| DC Severe | 0 | 0 | 0 | 0 | 0 | 0.9998 | 0.0002 |
| Death | 0 | 0 | 0 | 0 | 0 | 0 | 1 |
|  |  |  |  |  |  |  |  |
| **Cycle 5** | Mild | Moderate | Severe | DC Mild | DC Moderate | DC Severe | Death |
| Mild | 0.9644 | 0.0204 | 0.0150 | 0 | 0 | 0 | 0.0002 |
| Moderate | 0 | 0.9865 | 0.0133 | 0 | 0 | 0 | 0.0002 |
| Severe | 0 | 0.0057 | 0.9941 | 0 | 0 | 0 | 0.0002 |
| DC Mild | 0 | 0 | 0 | 0.9998 | 0 | 0 | 0.0002 |
| DC Moderate | 0 | 0 | 0 | 0 | 0.9998 | 0 | 0.0002 |
| DC Severe | 0 | 0 | 0 | 0 | 0 | 0.9998 | 0.0002 |
| Death | 0 | 0 | 0 | 0 | 0 | 0 | 1 |
|  |  |  |  |  |  |  |  |
| **Cycle 6** | Mild | Moderate | Severe | DC Mild | DC Moderate | DC Severe | Death |
| Mild | 0.9644 | 0.0204 | 0.0150 | 0 | 0 | 0 | 0.0002 |
| Moderate | 0 | 0.9865 | 0.0133 | 0 | 0 | 0 | 0.0002 |
| Severe | 0 | 0.0057 | 0.9941 | 0 | 0 | 0 | 0.0002 |
| DC Mild | 0 | 0 | 0 | 0.9998 | 0 | 0 | 0.0002 |
| DC Moderate | 0 | 0 | 0 | 0 | 0.9998 | 0 | 0.0002 |
| DC Severe | 0 | 0 | 0 | 0 | 0 | 0.9998 | 0.0002 |
| Death | 0 | 0 | 0 | 0 | 0 | 0 | 1 |
|  |  |  |  |  |  |  |  |
| **Cycle 65** | Mild | Moderate | Severe | DC Mild | DC Moderate | DC Severe | Death |
| Mild | 0.9643 | 0.0204 | 0.0150 | 0 | 0 | 0 | 0.0003 |
| Moderate | 0 | 0.9863 | 0.0133 | 0 | 0 | 0 | 0.0003 |
| Severe | 0 | 0.0057 | 0.9939 | 0 | 0 | 0 | 0.0003 |
| DC Mild | 0 | 0 | 0 | 0.9997 | 0 | 0 | 0.0003 |
| DC Moderate | 0 | 0 | 0 | 0 | 0.9997 | 0 | 0.0003 |
| DC Severe | 0 | 0 | 0 | 0 | 0 | 0.9997 | 0.0003 |
| Death | 0 | 0 | 0 | 0 | 0 | 0 | 1 |

**Table A3:** Transition probabilities for the Standard-of-Care arm of the model, based on the SAVANT trial used in scenario 1. DC = discontinuation with Sativex.

| **Cycle 1** | Mild | Moderate | Severe | DC Mild | DC Moderate | DC Severe | Death |
| --- | --- | --- | --- | --- | --- | --- | --- |
| Mild | 0.9998 | 0 | 0 | 0 | 0 | 0 | 0.0002 |
| Moderate | 0.2580 | 0.6450 | 0.0968 | 0 | 0 | 0 | 0.0002 |
| Severe | 0 | 0.1176 | 0.8822 | 0 | 0 | 0 | 0.0002 |
| DC Mild | 0 | 0 | 0 | 0.9998 | 0 | 0 | 0.0002 |
| DC Moderate | 0 | 0 | 0 | 0 | 0.9998 | 0 | 0.0002 |
| DC Severe | 0 | 0 | 0 | 0 | 0 | 0.9998 | 0.0002 |
| Death | 0 | 0 | 0 | 0 | 0 | 0 | 1 |
|  |  |  |  |  |  |  |  |
| **Cycle 2** | Mild | Moderate | Severe | DC Mild | DC Moderate | DC Severe | Death |
| Mild | 0.9998 | 0 | 0 | 0 | 0 | 0 | 0.0002 |
| Moderate | 0.1000 | 0.6999 | 0.2000 | 0 | 0 | 0 | 0.0002 |
| Severe | 0 | 0.1176 | 0.8822 | 0 | 0 | 0 | 0.0002 |
| DC Mild | 0 | 0 | 0 | 0.9998 | 0 | 0 | 0.0002 |
| DC Moderate | 0 | 0 | 0 | 0 | 0.9998 | 0 | 0.0002 |
| DC Severe | 0 | 0 | 0 | 0 | 0 | 0.9998 | 0.0002 |
| Death | 0 | 0 | 0 | 0 | 0 | 0 | 1 |
|  |  |  |  |  |  |  |  |
| **Cycle 3** | Mild | Moderate | Severe | DC Mild | DC Moderate | DC Severe | Death |
| Mild | 0.9998 | 0 | 0 | 0 | 0 | 0 | 0.0002 |
| Moderate | 0 | 0.9998 | 0 | 0 | 0 | 0 | 0.0002 |
| Severe | 0 | 0 | 0.9998 | 0 | 0 | 0 | 0.0002 |
| DC Mild | 0 | 0 | 0 | 0.9998 | 0 | 0 | 0.0002 |
| DC Moderate | 0 | 0 | 0 | 0 | 0.9998 | 0 | 0.0002 |
| DC Severe | 0 | 0 | 0 | 0 | 0 | 0.9998 | 0.0002 |
| Death | 0 | 0 | 0 | 0 | 0 | 0 | 1 |
|  |  |  |  |  |  |  |  |
| **Cycle 4** | Mild | Moderate | Severe | DC Mild | DC Moderate | DC Severe | Death |
| Mild | 0.9998 | 0 | 0 | 0 | 0 | 0 | 0.0002 |
| Moderate | 0 | 0.9998 | 0 | 0 | 0 | 0 | 0.0002 |
| Severe | 0 | 0 | 0.9998 | 0 | 0 | 0 | 0.0002 |
| DC Mild | 0 | 0 | 0 | 0.9998 | 0 | 0 | 0.0002 |
| DC Moderate | 0 | 0 | 0 | 0 | 0.9998 | 0 | 0.0002 |
| DC Severe | 0 | 0 | 0 | 0 | 0 | 0.9998 | 0.0002 |
| Death | 0 | 0 | 0 | 0 | 0 | 0 | 1 |
|  |  |  |  |  |  |  |  |
| **Cycle 5** | Mild | Moderate | Severe | DC Mild | DC Moderate | DC Severe | Death |
| Mild | 0.9998 | 0 | 0 | 0 | 0 | 0 | 0.0002 |
| Moderate | 0 | 0.9998 | 0 | 0 | 0 | 0 | 0.0002 |
| Severe | 0 | 0 | 0.9998 | 0 | 0 | 0 | 0.0002 |
| DC Mild | 0 | 0 | 0 | 0.9998 | 0 | 0 | 0.0002 |
| DC Moderate | 0 | 0 | 0 | 0 | 0.9998 | 0 | 0.0002 |
| DC Severe | 0 | 0 | 0 | 0 | 0 | 0.9998 | 0.0002 |
| Death | 0 | 0 | 0 | 0 | 0 | 0 | 1 |
|  |  |  |  |  |  |  |  |
| **Cycle 6** | Mild | Moderate | Severe | DC Mild | DC Moderate | DC Severe | Death |
| Mild | 0.9998 | 0 | 0 | 0 | 0 | 0 | 0.0002 |
| Moderate | 0 | 0.9998 | 0 | 0 | 0 | 0 | 0.0002 |
| Severe | 0 | 0 | 0.9998 | 0 | 0 | 0 | 0.0002 |
| DC Mild | 0 | 0 | 0 | 0.9998 | 0 | 0 | 0.0002 |
| DC Moderate | 0 | 0 | 0 | 0 | 0.9998 | 0 | 0.0002 |
| DC Severe | 0 | 0 | 0 | 0 | 0 | 0.9998 | 0.0002 |
| Death | 0 | 0 | 0 | 0 | 0 | 0 | 1 |
|  |  |  |  |  |  |  |  |
| **Cycle 65** | Mild | Moderate | Severe | DC Mild | DC Moderate | DC Severe | Death |
| Mild | 0.9997 | 0 | 0 | 0 | 0 | 0 | 0.0003 |
| Moderate | 0 | 0.9997 | 0 | 0 | 0 | 0 | 0.0003 |
| Severe | 0 | 0 | 0.9997 | 0 | 0 | 0 | 0.0003 |
| DC Mild | 0 | 0 | 0 | 0.9997 | 0 | 0 | 0.0003 |
| DC Moderate | 0 | 0 | 0 | 0 | 0.9997 | 0 | 0.0003 |
| DC Severe | 0 | 0 | 0 | 0 | 0 | 0.9997 | 0.0003 |
| Death | 0 | 0 | 0 | 0 | 0 | 0 | 1 |

**Table A4:** Transition probabilities for the Standard-of-Care + Sativex® arm of the model, based on the SAVANT trial for cycles 1-5 and the Arroyo et al. publication for cycles 6+ used in scenario 2. DC = discontinuation with Sativex.

| **Cycle 1** | Mild | Moderate | Severe | DC Mild | DC Moderate | DC Severe | Death |
| --- | --- | --- | --- | --- | --- | --- | --- |
| Mild | 0.2187 | 0.7186 | 0.0625 | 0 | 0 | 0 | 0.0002 |
| Moderate | 0.0937 | 0.7342 | 0.1718 | 0 | 0 | 0 | 0.0002 |
| Severe | 0.1000 | 0.1000 | 0.7998 | 0 | 0 | 0 | 0.0002 |
| DC Mild | 0 | 0 | 0 | 0.9998 | 0 | 0 | 0.0002 |
| DC Moderate | 0 | 0 | 0 | 0 | 0.9998 | 0 | 0.0002 |
| DC Severe | 0 | 0 | 0 | 0 | 0 | 0.9998 | 0.0002 |
| Death | 0 | 0 | 0 | 0 | 0 | 0 | 1 |
|  |  |  |  |  |  |  |  |
| **Cycle 2** | Mild | Moderate | Severe | DC Mild | DC Moderate | DC Severe | Death |
| Mild | 0.7694 | 0.0962 | 0.0962 | 0.0380 | 0 | 0 | 0.0002 |
| Moderate | 0.1002 | 0.7314 | 0.1302 | 0 | 0.0380 | 0 | 0.0002 |
| Severe | 0.0481 | 0.4328 | 0.4809 | 0 | 0 | 0.0380 | 0.0002 |
| DC Mild | 0 | 0 | 0 | 0.9644 | 0.0204 | 0.0150 | 0.0002 |
| DC Moderate | 0 | 0 | 0 | 0.0000 | 0.9865 | 0.0133 | 0.0002 |
| DC Severe | 0 | 0 | 0 | 0 | 0.0057 | 0.9941 | 0.0002 |
| Death | 0 | 0 | 0 | 0 | 0 | 0 | 1 |
|  |  |  |  |  |  |  |  |
| **Cycle 3** | Mild | Moderate | Severe | DC Mild | DC Moderate | DC Severe | Death |
| Mild | 0.9618 | 0 | 0 | 0.0380 | 0 | 0 | 0.0002 |
| Moderate | 0.3290 | 0.6328 | 0 | 0 | 0.0380 | 0 | 0.0002 |
| Severe | 0 | 0.2404 | 0.7213 | 0 | 0 | 0.0380 | 0.0002 |
| DC Mild | 0 | 0 | 0 | 0.9644 | 0.0204 | 0.0150 | 0.0002 |
| DC Moderate | 0 | 0 | 0 | 0 | 0.9865 | 0.0133 | 0.0002 |
| DC Severe | 0 | 0 | 0 | 0 | 0.0057 | 0.9941 | 0.0002 |
| Death | 0 | 0 | 0 | 0 | 0 | 0 | 1 |
|  |  |  |  |  |  |  |  |
| **Cycle 4** | Mild | Moderate | Severe | DC Mild | DC Moderate | DC Severe | Death |
| Mild | 0.9618 | 0 | 0 | 0.0380 | 0 | 0 | 0.0002 |
| Moderate | 0.1110 | 0.8508 | 0 | 0 | 0.0380 | 0 | 0.0002 |
| Severe | 0 | 0 | 0.9618 | 0 | 0 | 0.0380 | 0.0002 |
| DC Mild | 0 | 0 | 0 | 0.9644 | 0.0204 | 0.0150 | 0.0002 |
| DC Moderate | 0 | 0 | 0 | 0 | 0.9865 | 0.0133 | 0.0002 |
| DC Severe | 0 | 0 | 0 | 0 | 0.0057 | 0.9941 | 0.0002 |
| Death | 0 | 0 | 0 | 0 | 0 | 0 | 1 |
|  |  |  |  |  |  |  |  |
| **Cycle 5** | Mild | Moderate | Severe | DC Mild | DC Moderate | DC Severe | Death |
| Mild | 0.8444 | 0.1206 | 0 | 0.0348 | 0 | 0 | 0.0002 |
| Moderate | 0.2412 | 0.7237 | 0 | 0 | 0.0348 | 0 | 0.0002 |
| Severe | 0 | 0 | 0.9650 | 0 | 0 | 0.0348 | 0.0002 |
| DC Mild | 0 | 0 | 0 | 0.9644 | 0.0204 | 0.0150 | 0.0002 |
| DC Moderate | 0 | 0 | 0 | 0 | 0.9865 | 0.0133 | 0.0002 |
| DC Severe | 0 | 0 | 0 | 0 | 0.0057 | 0.9941 | 0.0002 |
| Death | 0 | 0 | 0 | 0 | 0 | 0 | 1 |
|  |  |  |  |  |  |  |  |
| **Cycle 6+** | Mild | Moderate | Severe | DC Mild | DC Moderate | DC Severe | Death |
| Mild | 0.9644 | 0.0204 | 0.0150 | 0 | 0 | 0 | 0.0002 |
| Moderate | 0 | 0.9865 | 0.0133 | 0 | 0 | 0 | 0.0002 |
| Severe | 0 | 0.0057 | 0.9941 | 0 | 0 | 0 | 0.0002 |
| DC Mild | 0 | 0 | 0 | 0.9998 | 0 | 0 | 0.0002 |
| DC Moderate | 0 | 0 | 0 | 0 | 0.9998 | 0 | 0.0002 |
| DC Severe | 0 | 0 | 0 | 0 | 0 | 0.9998 | 0.0002 |
| Death | 0 | 0 | 0 | 0 | 0 | 0 | 1 |
|  |  |  |  |  |  |  |  |
| **Cycle 65** | Mild | Moderate | Severe | DC Mild | DC Moderate | DC Severe | Death |
| Mild | 0.9643 | 0.0204 | 0.0150 | 0 | 0 | 0 | 0.0003 |
| Moderate | 0 | 0.9863 | 0.0133 | 0 | 0 | 0 | 0.0003 |
| Severe | 0 | 0.0057 | 0.9939 | 0 | 0 | 0 | 0.0003 |
| DC Mild | 0 | 0 | 0 | 0.9997 | 0 | 0 | 0.0003 |
| DC Moderate | 0 | 0 | 0 | 0 | 0.9997 | 0 | 0.0003 |
| DC Severe | 0 | 0 | 0 | 0 | 0 | 0.9997 | 0.0003 |
| Death | 0 | 0 | 0 | 0 | 0 | 0 | 1 |
